# New ecofriendly heterogeneous nano-catalyst for the synthesis of 1-substituted and 5-substituted 1*H*-tetrazole derivatives

**DOI:** 10.1038/s41598-022-19478-w

**Published:** 2022-09-13

**Authors:** Mahboobeh-Sadat Mashhoori, Reza Sandaroos

**Affiliations:** Department of Chemistry, Faculty of Science, University of Birjand, P.O. Box 97175-615, Birjand, Iran

**Keywords:** Chemistry, Materials science

## Abstract

A novel ecofriendly heterogeneous catalyst containing Schiff base coordinated Cu(II) covalently attached to Fe_3_O_4_@SiO_2_ nanoparticles through imidazolium linker [Fe_3_O_4_@SiO_2_-Im(Br)-SB-Cu (II)] was synthesized and characterized by using various techniques. The catalytic efficiency of this nano-catalyst was tested in water in the synthesis of tetrazole derivatives using two one-pot multicomponent reaction (MCR) models: The synthesis of 1-aryl 1*H*-tetrazole derivatives from the reaction of aniline, triethyl orthoformate, and sodium azide and the synthesis of 5-aryl 1*H*-tetrazole derivatives from the reaction of benzaldehyde, hydroxy amine hydrochloride, and sodium azide. The investigation showed that (i) The catalyst is highly efficient in the synthesis of tetrazole derivatives with high yield (97%) in aqueous medium and mild temperatures; (ii) The catalytic effectiveness is due to the synergy between the metallic center and the imidazolium ion and (iii) The reuse advantage of the catalyst without contamination or significant loss (12% of loss range) in the catalytic activity.

## Introduction

Tetrazoles are essential class of poly-aza-heterocyclic compounds largely discovered in nature^1^. Recently, tetrazoles have received much attention due to their large spectrum of applications in the field of medicine and biology such as anticancer, antiviral, antiallergic, antibiotic, anti-HIV, etc.,^2–5^. Homogeneous catalysts were predominantly used, due to their solubility and high activity, in the synthesis of tetrazole derivatives than their heterogeneous catalysts counterpart. However, homogeneous catalysts suffer from many drawbacks such as high temperature working conditions, difficult recycling, product contamination, and deactivation through dimerization. To overcome these problems, methods were devised to heterogenized such catalysts by grafting them on organic^6^ and inorganic supports^7–9^, including polymerization^10,11^. Considering drawbacks associated with both homogenous and heterogeneous catalyst systems, researcher dedicated considerable efforts to develop efficient synthetic methodologies for the synthesis of tetrazole derivatives using nanostructured catalyst systems. Since 2010, ample nano-catalysts or nanomaterials supported catalysts have been explored to avoid drawbacks associated with the conventional strategies for the synthesize of tetrazole derivatives^12–17^. A review published by Mittal and Awasthi^12^ summarized the most important Nano-based catalyst strategies used in the synthesis of 5-substituted 1*H*-tetrazole derivatives. Examples of useful nano-catalyst strategies such as the use of Fe_3_O_4_ NPs described by Kolo and Sajadi^13^, the use of 4′-phenyl-2,2′:6′,2′′-terpyridine–copper(II) complex immobilized onto activated multi- walled carbon nanotubes [AMWCNTs-O-Cu(II)-PhTPY] reported by Sharghi and co-workers^14^, salen complex of Cu(II) supported on superparamagnetic Fe_3_O_4_@SiO_2_ nanoparticles [Fe_3_O_4_@SiO_2_/Salen complex of Cu(II)] reported by Sardarian and co-workers^15^, ligand complex, including Schiff base, of copper(II) supported on superparamagnetic Fe_3_O_4_@SiO_2_ nanoparticles used by Javidi and co-workers^16^ and Cu/aminoclay/reduced graphene oxide nanohybrid (Cu/AC/r-GO nano-hybrid) reported by Soltan Rad and co-workers^17^ were developed for the preparation of various 5-substituted tetrazole derivatives to have all the possible benefits and advantages in terms of performance, reusability, and ease of use. However, most of the strategies summarized in this review require solvent-free conditions at high temperatures or solvents at high temperatures which can be a limitation for their applications. Magnetic nanoparticles, especially the cost effective and well-studied Fe_3_O_4_^18–20^, have number of remarkable features like high active surface area, low toxicity, superparamagnetism^21^, ease of recycling due to their removal from reaction mixtures with an external magnet^22,23^, high dispersion and reactivity, and chemical/thermal stability. Also, ease of surface modification and ligands coupling due to the chemical nature and accessible reactive groups on the surface of the nanoparticles^24^. All these characteristics make them very attractive as an ideal support for nano-catalyst systems for the synthesis of tetrazole derivatives.

The idea of green chemistry has become an integral part of sustainability which makes catalysis science even more innovative. From the viewpoint of green chemistry, sustainable catalyst must possess a series of distinct advantages like high activity, selectivity and efficiency, reasonable recovery, high stability, and excellent recyclability. To address these requirements, magnetic nano particles have received tremendous attention in the last decades as an excellent catalyst supporting materials for the synthesis of tetrazole derivatives. Recent advances in this area were summarized in the well documented review by Rahul Shrivastava and co-workers^25^. For the synthesis of 5-substituted tetrazole via [3 + 2] cycloaddition reaction, two interesting reports by Tamoradi and co-workers, described the use of magnetic Fe_3_O_4_@tryptophan–La and Fe_3_O_4_@tryptopahn-Nd in water @ 80 °C^26,27^, Fe_3_O_4_-adenine-Zn was used in PEG 400 at 120 °C^28^ Fe_3_O_4_@tryptophan@Ni was reported to work in PEG 400 at 120 °C and a palladium(0) based nano-catalyst, Fe_3_O_4_@l-lysine-Pd(0), was described by Ashraf and co-workers to work in water at 100 °C^29^. For the synthesis of 1-substituted tetrazole derivatives via multicomponent reaction (MCR), Habibi and co-workers have reported the use of Fe_3_O_4_@5,10-dihydropyrido[2,3-*b*]quinoxaline-7,8-diol copper and Fe_3_O_4_@1,10-phenanthroline-5,6-diol@Mn complexes in the reaction between amine, ethyl orthoformate and sodium azide under solvent-free conditions^30,31^ A bifunctional magnetite nano-catalyst (Fe_3_O_4_/HT-NH_2_-Cu^II^) was used by Salimi and Zamanpour group in the reaction between aromatic amines, ethyl orthoformate and sodium azide in water @ 90 °C^32^, and Salimi and co-workers also reported the use of Fe_3_O_4_@HT@AEPH_2_-Co^II^ in the same MCR between amine, ethyl orthoformate and solidum azide in water at 90 °C^33^. Due to the broad spectrum of tetrazole derivatives applications, there is still a growing need of innovative green and ecofriendly catalytic systems for the synthesis of tetrazole derivatives. With this idea in mind, we embarked in the development of new Fe_3_O_4_ supported nano-catalyst that is suitable to work in aqueous conditions with all the benefits and advantages of a magnetic nano-catalyst. To allow the catalyst to work in water, we based our design on the use of water-soluble linker or coupling spacer arm to bring the catalyst to aqueous medium during the catalytic process. We decided to focus on a well-studied, cost effective and tested Cu(II)-coordinated Schiff base nano-catalyst having an imidazolium linker to nanoparticles. The nano-catalyst efficiency was then successfully investigated in one-pot multicomponent reaction (MCR) synthesis of tetrazole derivatives in aqueous medium. The catalyst achieved high tetrazole derivatives yield in a short reaction time and, due to its magnetic characteristic, it was easily removed from the products without leaving behind any metallic contamination.

## Experimental

### Materials and apparatus

All solvents were purchased from Merck Co. and dried by standard procedures. All chemical reagents were purchased from Sigma-Aldrich chemical company and used without further purification. The progress of reactions was monitored by TLC on Silica-gel Polygram SILG/UV254 plates. Fourier Transform Infrared (FT-IR) spectra were recorded on a PerkinElmer 780 FT-IR spectrometer (KBr tablets). The morphology (SEM) and elemental analysis (EDS) of the catalyst were determined by using the FE-SEM TESCAN MIRA3 instrument. Transmission Electron Microscopy (TEM) images were obtained with a Philips EM208 S electron microscope. X‐ray Diffraction (XRD) patterns were collected using a Philips PW 1730 diffractometer using Cu Kα radiation (λ = 1.54 A°). Thermogravimetric Analysis (TGA) was performed on a Q600 TA instrument at 30–700 °C with a heating rate of 20 °C min^−1^ in an argon atmosphere. Vibrating Sample Magnetometer (VSM) analysis was performed at room temperature using an LBKFB instrument. Inductively Coupled Plasma-Optical Emission Spectroscopy (ICP-OES) analysis was performed using a Simultaneous VISTA-PRO instrument. Atomic Absorption Spectroscopy (AAS) analysis was performed using a Shimadzu AA6200 instrument.

### The synthesis of modified silica coated Fe_3_O_4_ nanoparticles (Fe_3_O_4_@SiO_2_)

The silica coated Fe_3_O_4_ magnetic nanoparticles were synthesized by previously reported methods^34^. FeCl_3_.6H_2_O (6.8 g) and FeCl_2_.4H_2_O (2.5 g) were added to deionized water (300 mL) and stirred under nitrogen gas at room temperature. Gradually, ammonia solution (25% w/w, 70 mL) was added to the vigorously stirred mixture. As soon as the solution's color turned black, the resulting nanoparticles were separated by an external magnet and washed several times with deionized water.

To synthesize silica-coated nanoparticles, Fe_3_O_4_ nanoparticles (3.0 g) were dispersed by sonication in a deionized water/ethanol solvent mixture (1:4 v/v, 500 mL) 30 min. Then a solution of ammonia (25% w/w) was gradually added until the pH reaches 10. The tetraethyl orthosilicate (TEOS, 20 mL) was slowly added to the mixture and stirred three hours at 50 °C. The silica-coated nanoparticles (Fe_3_O_4_@SiO_2_) were collected by a permanent magnet and washed with deionized water and ethanol several times and dried in a vacuum oven at 50 °C for 24 h. In the final stage, Fe_3_O_4_@SiO_2_ (1 g) was sonicated in dry toluene (40 mL) for 30 min. Then, 3-Chloropropyl triethoxysilane (2.0 mL) was added dropwise and refluxed for 20 h. The resulting chloro-modified Fe_3_O_4_@SiO_2_ was removed from the reaction mixture by a strong magnet, washed with in toluene, ethanol and diethyl ether for several times. Then dried under vacuum at 60 °C for 12 h^35^. The loading amount of Cl atom was 0.3 mmol per gram catalyst based on EDX.

### The synthesis of Fe_3_O_4_@ SiO_2_-Im nanoparticles

Imidazole (0.5 mmol, 0.034 g) was added to the dispersed solution of chloro-modified Fe_3_O_4_@SiO_2_ (1.0 g) in dry toluene (40 mL) and triethylamine (NEt_3_, 0.5 mmol, 0.05 g) was added dropwise and refluxed for 24 h. The resulting nanoparticles were separated with the external magnet and washed with distilled water and ethanol. The resulted Fe_3_O_4_@ SiO_2_-Im nanoparticles were dried in a vacuum oven at 80 °C for 12 h.

### The synthesis of Fe_3_O_4_@ SiO_2_-Im[Br] nanoparticles

The synthesized nanoparticles of Fe_3_O_4_@SiO_2_-Im (1.0 g) in dry ethanol (40 mL) were dispersed for 30 min by sonication. The ethanolic solution of 3-Bromopropylamin hydrobromide (0.5 mmol, 0.11 g) was gradually added to the stirred mixture and refluxed for 48 h. The resulting nanoparticles were separated by the magnet, washed with distilled water, ethanol, and diethyl ether. Finally, nanoparticles were dried in a vacuum at 80 °C for 20 h.

### The synthesis of Fe_3_O_4_@ SiO_2_-Im[Br]-SB nanoparticles

The nanoparticles (Fe_3_O_4_@ SiO_2_-Im [Br]-PrNH_2_. HBr, 1.0 g) from the previous step were dispersed by sonication in dry ethanol (40 mL) followed by dropwise addition of salicylaldehyde (0.5 mmol, 56 µL) and sodium hydroxide (NaOH, 0.5 mmol, 0.02 g) solutions. The reaction mixture was then refluxed in ethanol 20 h. The resulting nanoparticles were separated by the magnet and washed with distilled water, ethanol, and diethyl ether. Finally, nanoparticles were dried in a vacuum at 80 °C for 20 h.

### The synthesis of Fe_3_O_4_@ SiO_2_-Im[Br]-SB-Cu (II) Nano-complex

The ethanolic solution of Cu (OAC)_2_. H_2_O (0.8 mmol, 0.16 g) was added dropwise to the well dispersed Fe_3_O_4_@ SiO_2_-Im [Br]-Schiff base nanoparticles (1.0 g)in ethanol and refluxed 12 h. The resulting Cu(II)-coordinated nanoparticles were separated by an external magnet and washed several times with ethanol, diethyl ether and dried in a vacuum at 60 °C for 10 h.

### General procedure for the synthesis of 1-substituted 1H-tetrazole

aniline (1.0 mmol, 0.09 mL), Triethyl orthoformate (1.2 mmol, 0.2 mL), sodium azide (1.0 mmol, 0.06 g) in a water (1.0 mL) in the presence of Fe_3_O_4_@SiO_2_-Im[Br]-SB-Cu (II) nano-catalyst (0.6 mol%, 0.008 g) were stirred at 40 °C. The reaction progression was monitored by thin-layer chromatography (TLC) at different interval of time using n-Hexane/Ethyl acetate (4:1) as eluent. At the end, the reaction mixture was cooled, and catalyst removed by an external magnet. The reaction mixture was extracted with 3 × 10 mL of ethyl acetate. The organic phase was dried with anhydrous Na_2_SO_4_, filtered and then evaporated. The pure product was obtained by recrystallization in a mixture of n-Hexane/Ethyl acetate. The recovered yield was 97%.

### General procedure for the synthesis of 5-substituted 1H-tetrazole

Benzaldehyde (1.0 mmol, 0.1 mL), hydroxylammonium chloride (1.0 mmol, 0.07 g), sodium azide (1.2 mmol, 0.08 g) in water (1.0 mL) in the presence of Fe_3_O_4_@ SiO_2_-Im[Br]-SB-Cu (II) nano-catalyst (0.9 mol%, 0.012 g) were stirred at 40 °C. The reaction was followed by thin-layer chromatography (TLC) at different time intervals in (n-Hexane/Ethyl acetate: 4:1). The reaction mixture was cooled, and the catalyst removed by an external magnet. 5 mL of HCl (5 N) was added to the reaction mixture and extracted with 3 × 10 mL of ethyl acetate. The organic phase was extracted again with the HCl solution (1 N), followed by a saturated solution of NaCl. The organic phase was dried with the anhydrous Na_2_SO_4_, filtered and then evaporated. The pure product was obtained in 97% yield by recrystallization with n-Hexane/Ethyl acetate solvent mixture.

## Results and discussion

### Catalyst characterization

**Figure 1:**
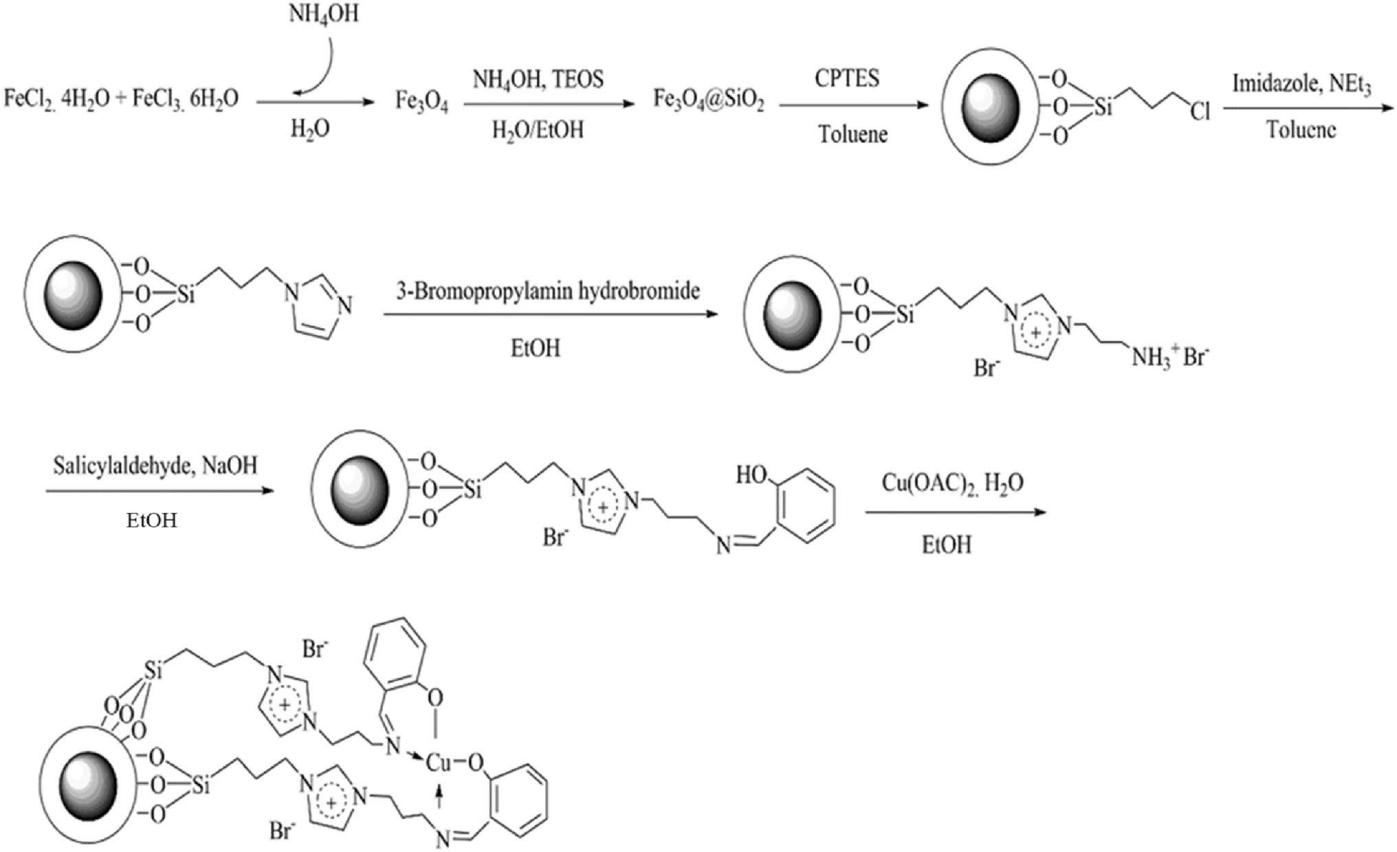
Synthesis of Fe_3_O_4_@SiO_2_-Im[Br]-SB-Cu (II) nano-catalyst.

**Figure 2:**
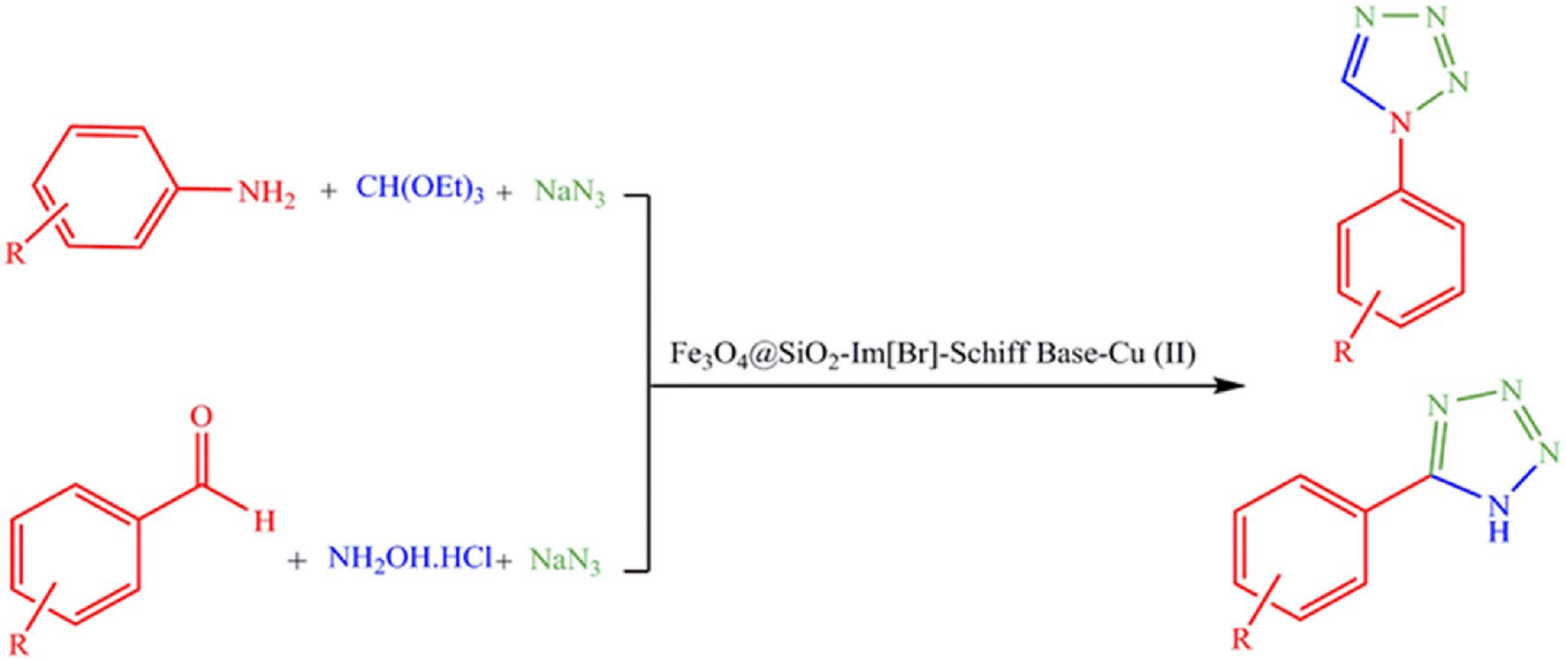
Synthesis of the tetrazole derivatives in the presence Fe_3_O_4_@SiO_2_-Im[Br]-SB-Cu (II) nano-catalyst.

**Figure 3:**
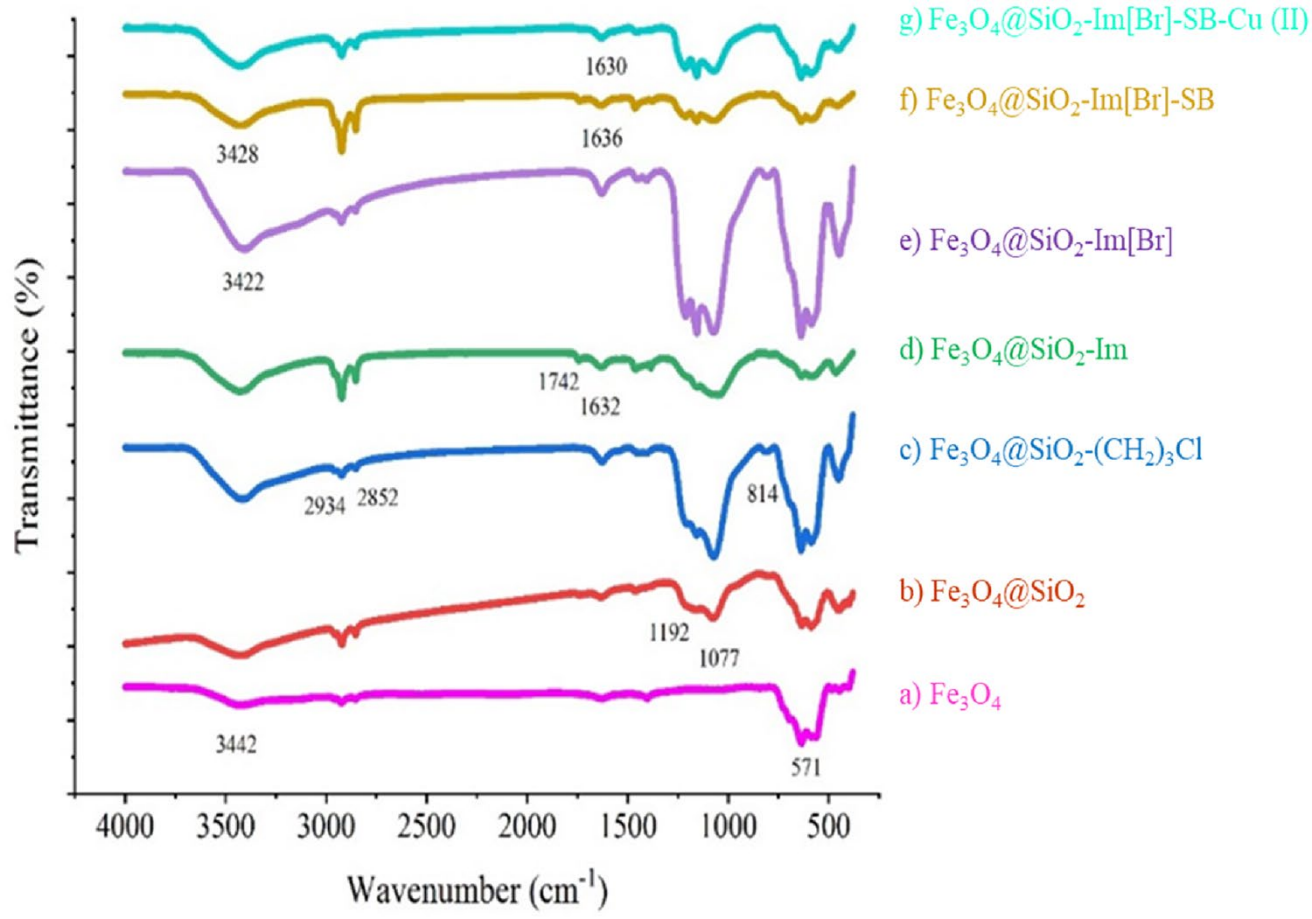
FT-IR spectra: (**a**) Fe_3_O_4_; (**b**) Fe_3_O_4_@SiO_2_; (**c**) Fe_3_O_4_@SiO_2_-(CH_2_)_3_Cl; (**d**) Fe_3_O_4_@SiO_2_-Im; (**e**) Fe_3_O_4_@SiO_2_-Im[Br]; (**f**) Fe_3_O_4_@SiO_2_-Im[Br]-SB; (**g**) Fe_3_O_4_@SiO_2_-Im[Br]-SB-Cu (II).

Fe_3_O_4_@SiO_2_-Im[Br]-SB-Cu(II) nano-catalyst was synthesized as depicted in Fig. 1 and investigated in the synthesis of tetrazole derivatives (Fig. 2). The prepared catalyst is characterized by various methods. The FT-IR spectra of the catalyst synthesis steps are shown in Fig. 3. The spectrum 1a, which corresponds to the Fe_3_O_4_ nanoparticles, show the peaks at 571 and 3442 cm^−1^ corresponding to stretching vibrations of Fe–O and OH groups, respectively^36,37^. The appearance of new peaks at 1077 and 1192 cm^−1^ are corresponding to Si–O (Symm.) and Si–O (Asymm.), respectively. These peaks are a confirmation that the surface of nanoparticles is protected by silica coating layer (Fig. 3, 1b)^38^. The transmittance of core shelled Fe_3_O_4_ nanoparticles was slightly lower than that of Fe_3_O_4_ nanoparticles due to silica coating. Absorbed peaks in 2852 (Symm.), 2934 (Asymm.), 1420 (Bending) and 814 cm^−1^, respectively, correspond to CH_2_ and C–Cl are evidence of the modification of nanoparticles surface (Fig. 3, 1c)^39^. The disappearing C–Cl peak and the appearance of new peaks in 1632 and 1742 cm^−1^ indicate that the imidazole ring was coupled to the nanoparticles surface (Fig. 3, 1d)^40^. The spectrum 1e shows peak in 3422 cm^−1^, which correspond to NH of amine group. The new peak at 1636 cm^−1^ is evidence of the formation of imine (Fig. 3, 1f)^41^. The new peaks at 635 and 620 cm^−1^ correspond to Cu–N and Cu–O. Also, the transfer of imine peak to lower frequencies confirms the formation of the metal complex (Fig. 3, 1g).

**Figure 4:**
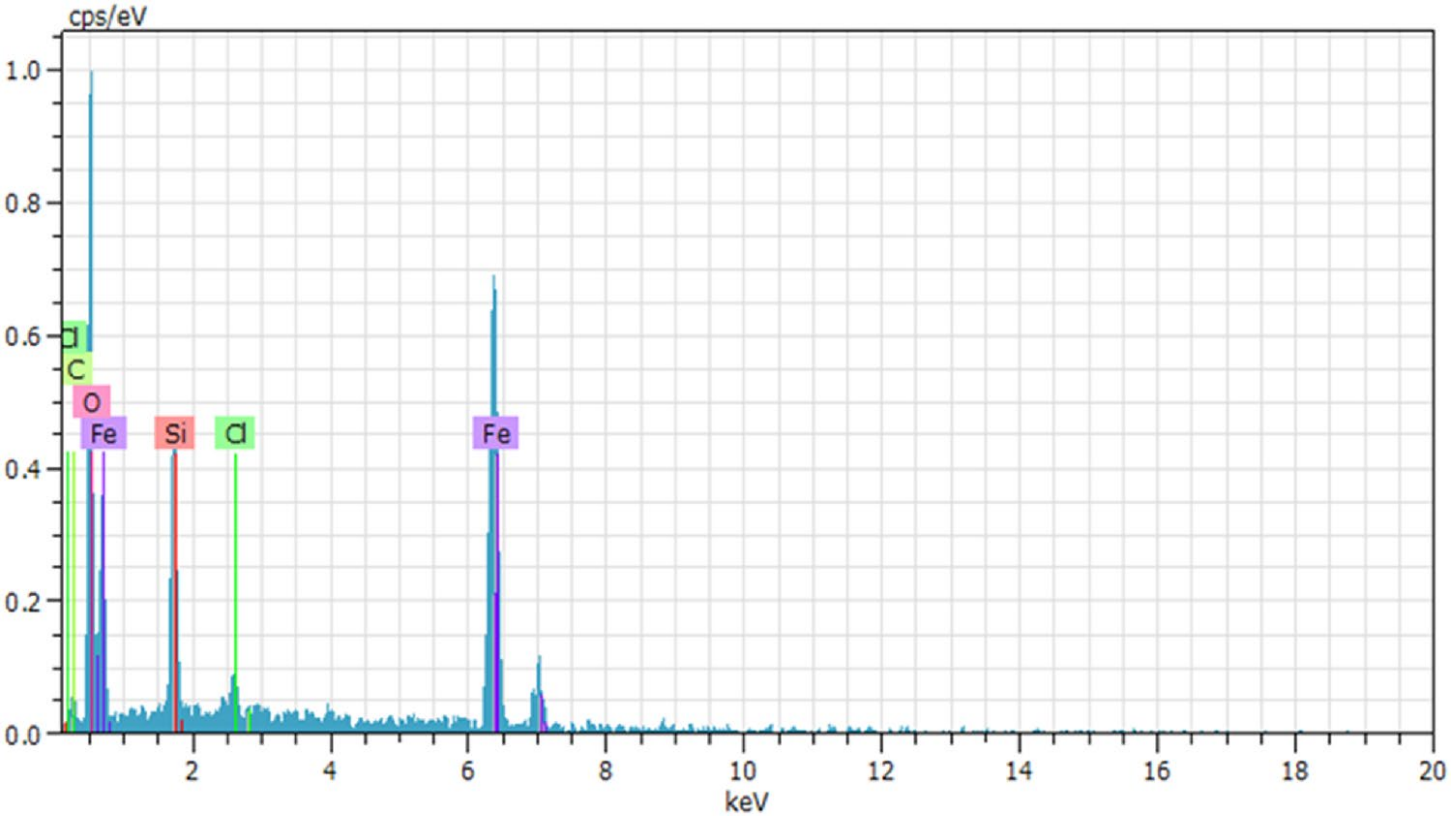
EDX Spectrum of Fe_3_O_4_@SiO_2_-Cl.

**Figure 5:**
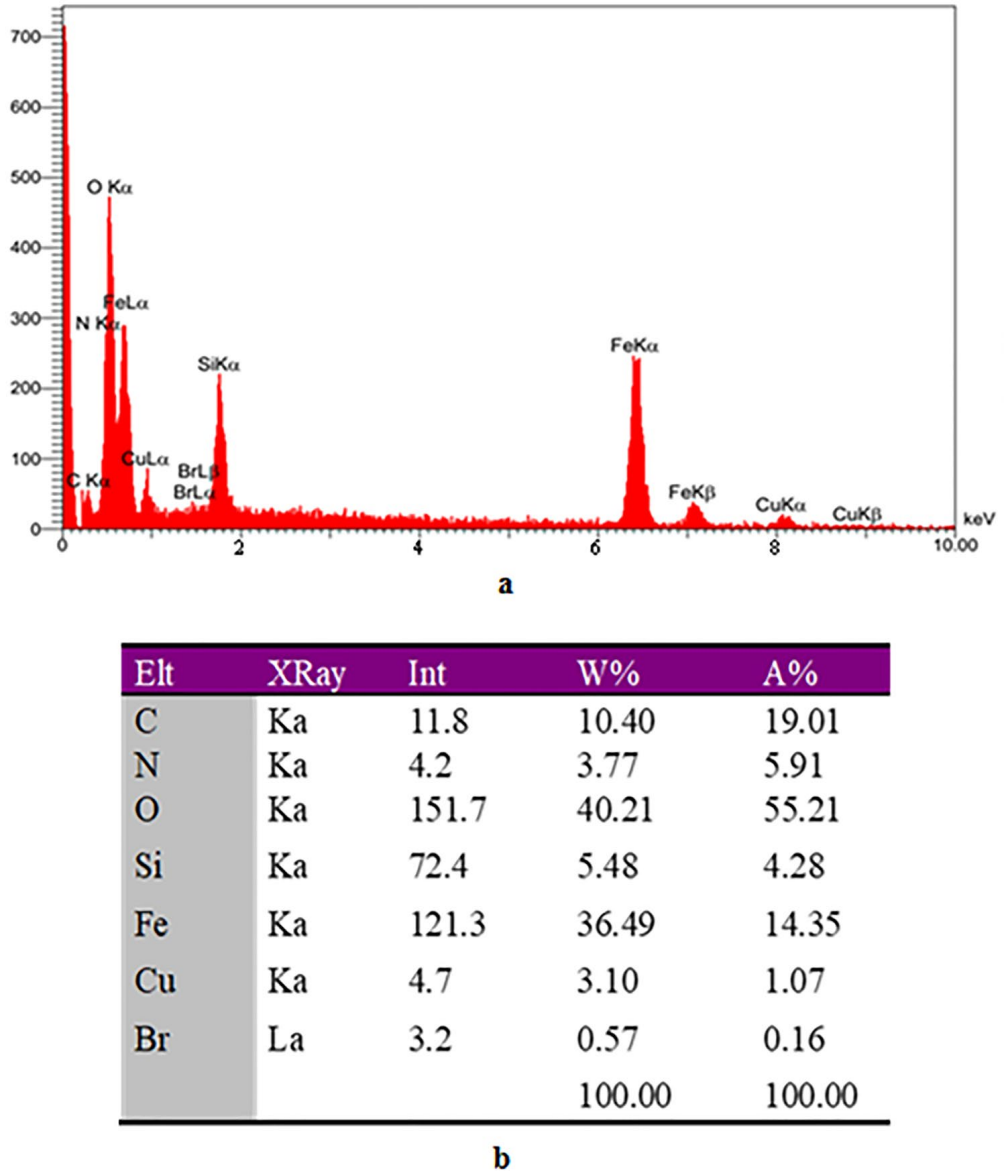
(**a**) EDX Spectrum and (**b**) Projected elemental composition of Fe_3_O_4_@SiO_2_-Im[Br]-SB-Cu (II).

The elemental composition of Fe_3_O_4_@SiO_2_-Cl (Fig. 4) and the nano-catalyst Fe_3_O_4_@SiO_2_-Im[Br]-SB-Cu (II) (Fig. 5) was determined by Energy Dispersive X-rays (EDX) analysis. Absence of chlorine element in the nano-catalyst confirms the attachment of ionic metal Schiff base complex on the surface of modified nanoparticles leading to the nano-catalyst. Anticipated elemental composition: C (10.40%), N (3.77%), O (40.21%), Si (5.48%), Fe (36.49%), Br (0.57%) and Cu (3.10%).

**Figure 6:**
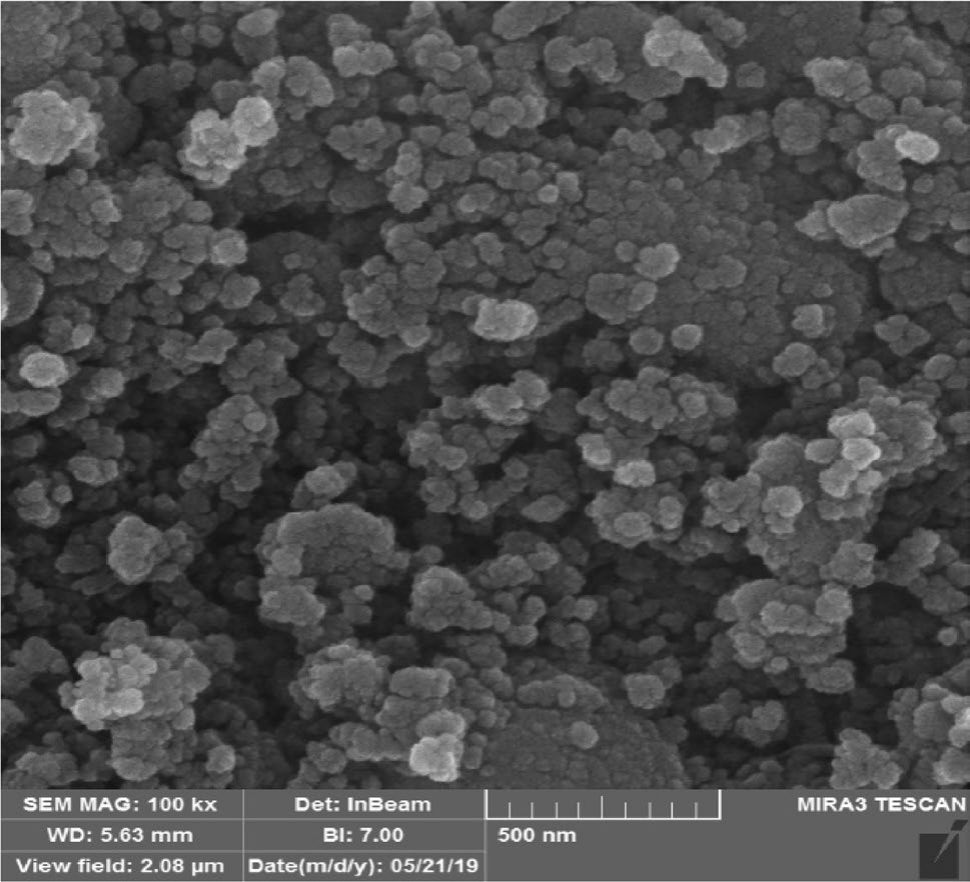
SEM image of Fe_3_O_4_@SiO_2_-Im[Br]-SB-Cu (II) nano-catalyst.

The morphology of Fe_3_O_4_@SiO_2_-Im[Br]-SB-Cu (II) nano-catalyst was determined by Scanning Electron Microscopy (SEM). SEM images show spherical and irregular shapes for the nanoparticles (Fig. 6).

**Figure 7:**
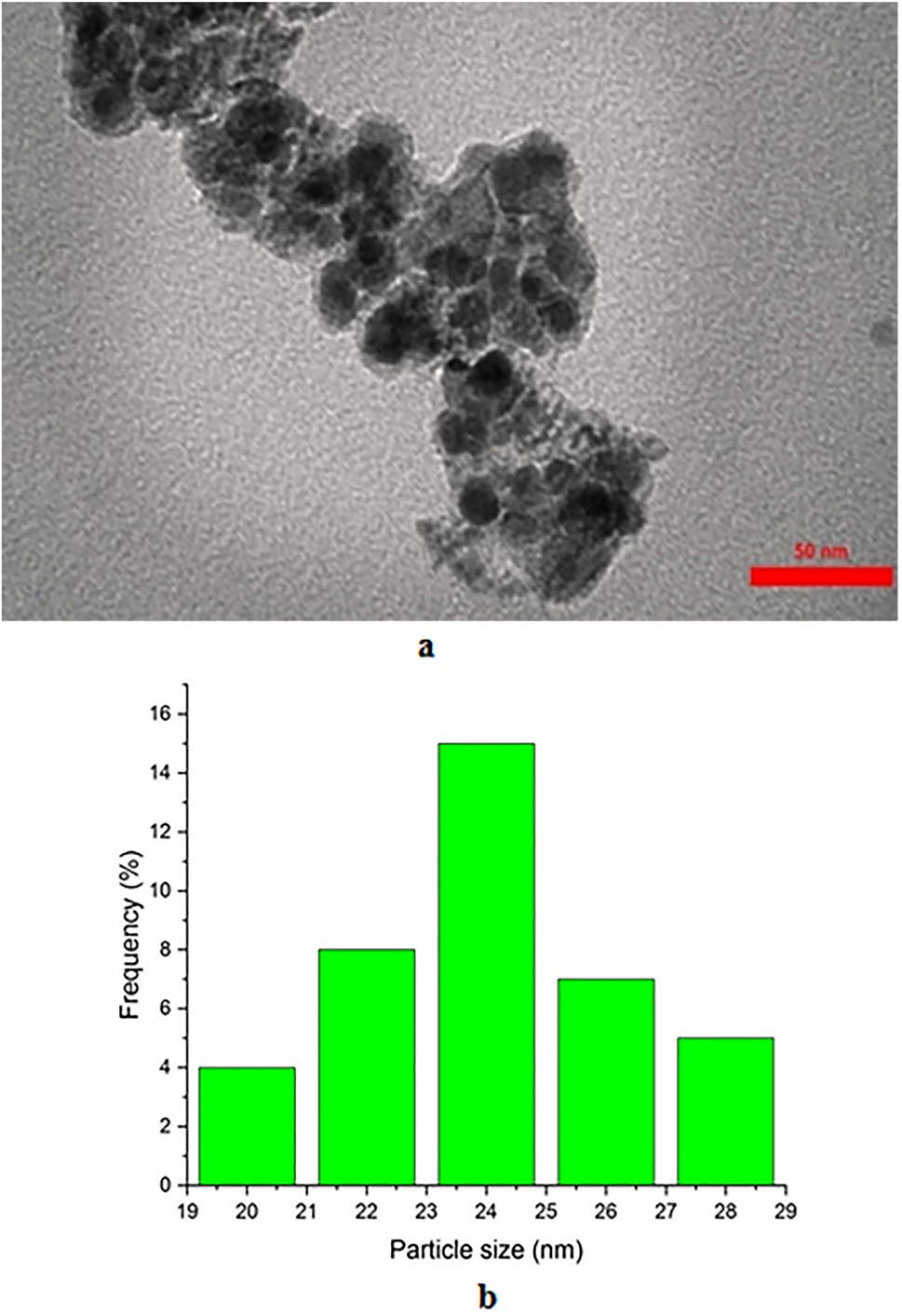
(**a**) TEM image (scale bar at 50 nm) and (**b**) histogram of Fe_3_O_4_@SiO_2_-Im[Br]-SB-Cu (II) nano-catalyst.

The morphology of Fe_3_O_4_@SiO_2_-Im[Br]-SB-Cu (II) nano-catalyst was also determined by Transmission electron microscopy (TEM) (Fig. 7a). Also, according to the histogram diagram of the nano-catalyst, the average particle size was estimated to be about 24 nm (Fig. 7b).

**Figure 8:**
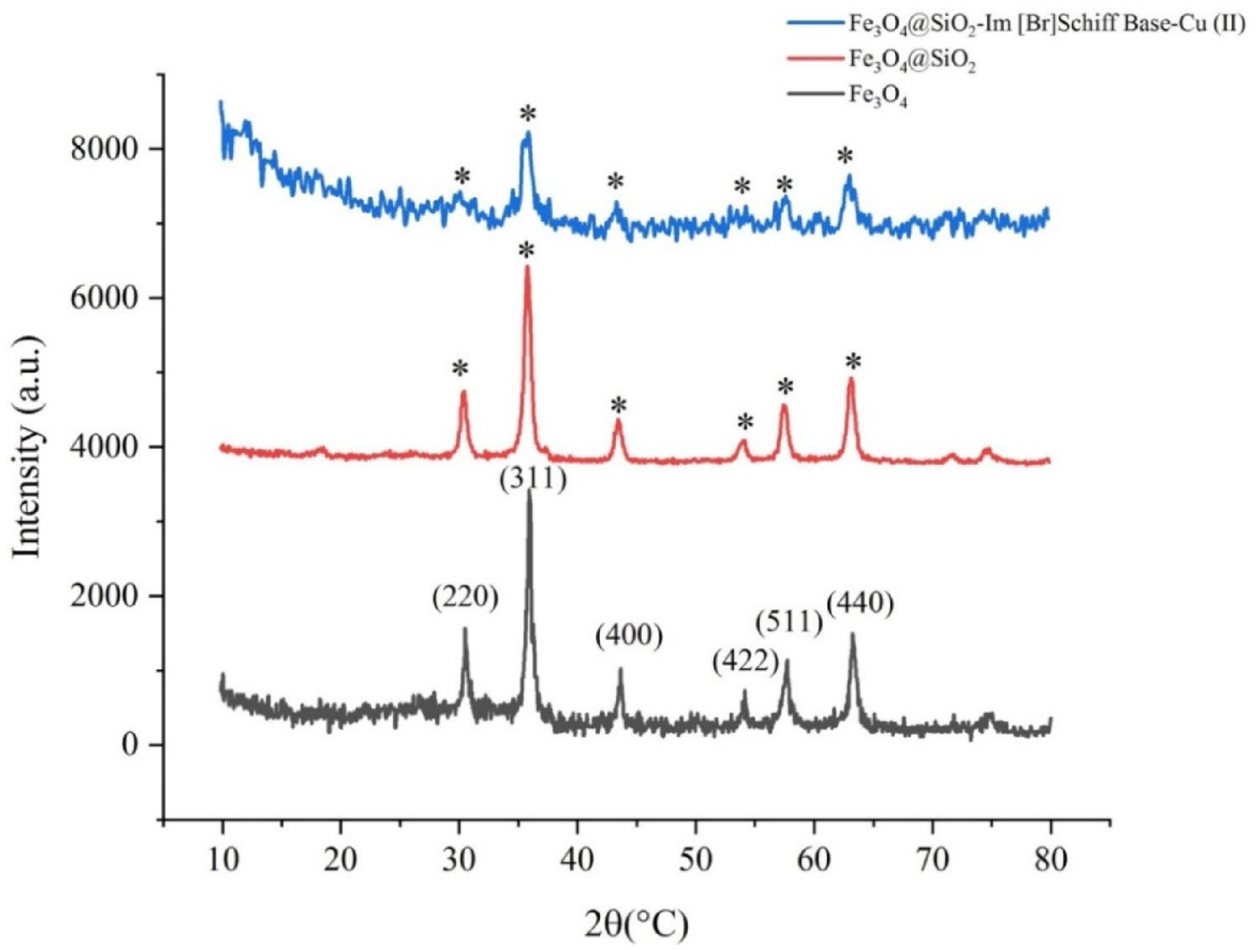
XRD patterns of Fe_3_O_4_, Fe_3_O_4_@SiO_2_ and Fe_3_O_4_@SiO_2_-Im[Br]-SB-Cu (II) nano-catalyst.

The X-ray diffraction (XRD) patterns of Fe_3_O_4_, Fe_3_O_4_ @ SiO_2_ and Fe_3_O_4_@SiO_2_-Im [Br] -SB-Cu (II) are shown in Fig. 8. The XRD pattern of Fe_3_O_4_ magnetic nanoparticles is in accordance with (PDF # 88-0866, reference JCPDS card no. 19-629), which shows a crystalline cubic spinel structure^42^. XRD patterns of Fe_3_O_4_, Fe_3_O_4_ @ SiO_2_ and Fe_3_O_4_ @ SiO_2_-Im[Br]-SB-Cu (II) show the peaks in 2θ = 30.1, 35.4, 43.1, 53.4, 57, and 62.6° which are related to the phase of (220), (311), (400), (422), (511), and (440), respectively and are in full agreement with the Fe_3_O_4_ pattern showing that their crystalline phase and position have not changed. These results indicate that the crystalline cubic structure of nanoparticles Fe_3_O_4_ is preserved during the catalyst preparation process. In the Fe_3_O_4_@SiO_2_ spectrum, a broad peak is observed in 2θ = 10–20°, which is related to amorphous silica. This broad peak for the nano-catalyst was shifted to lower angles due to the synergetic effect of amorphous silica and Cu(II)-coordinated Schiff base. The average size of nanoparticles was calculated by the Debye–Scherrer equation (D = K.λ/β.cosθ, λ (wavelength, 0.154 nm), K (a crystallized form factor, 0.94), β (Full width at half maximum, (rad)), θ (Bragg reflection angle, (°)) to be about 28 nm which correspond to the TEM results.

**Figure 9:**
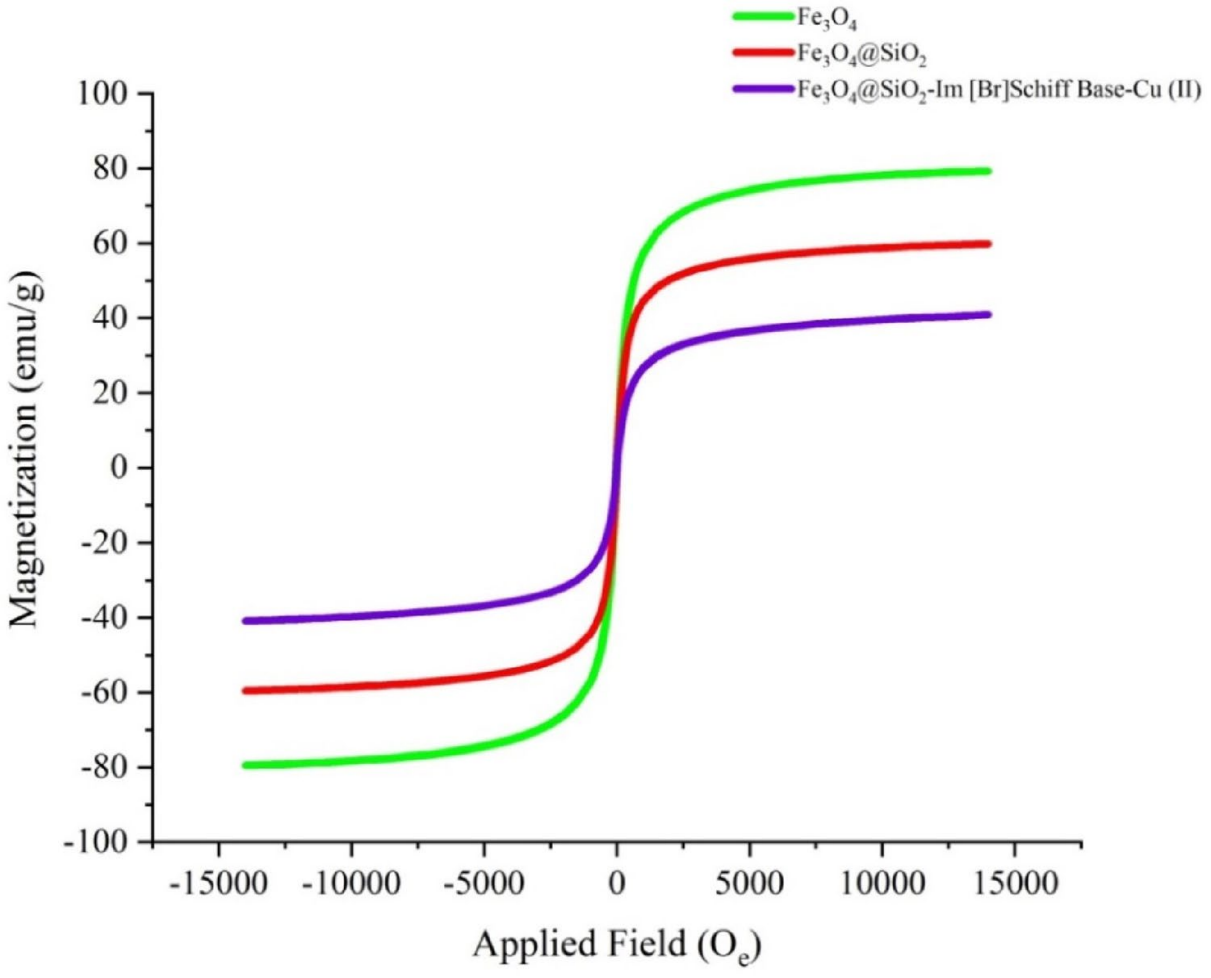
Magnetization curves of Fe_3_O_4_, Fe_3_O_4_@SiO_2_ and Fe_3_O_4_@SiO_2_-Im[Br]-SB-Cu (II) nano-catalyst.

The magnetic property of nano-catalyst was measured at different steps of the synthesis by vibrating sample magnetometer (VSM) (Fig. 9). As shown in Fig. 9, the magnetic properties of nanoparticles are gradually reduced by the silica layer coating and by the coupling of the Cu(II)-complex to the surface of the nanoparticles. Although the magnetic saturation values for Fe_3_O_4_, Fe_3_O_4_@SiO_2_ and Fe_3_O_4_@SiO_2_-Im[Br]-SB-Cu (II) are 80, 58 and 38 emu g^-1^, respectively, nano-catalyst has still a strong magnetic property for its removal from the reaction mixture by an external magnet. This was confirmed by the catalyst recycling study (see Fig. 15).

**Figure 10:**
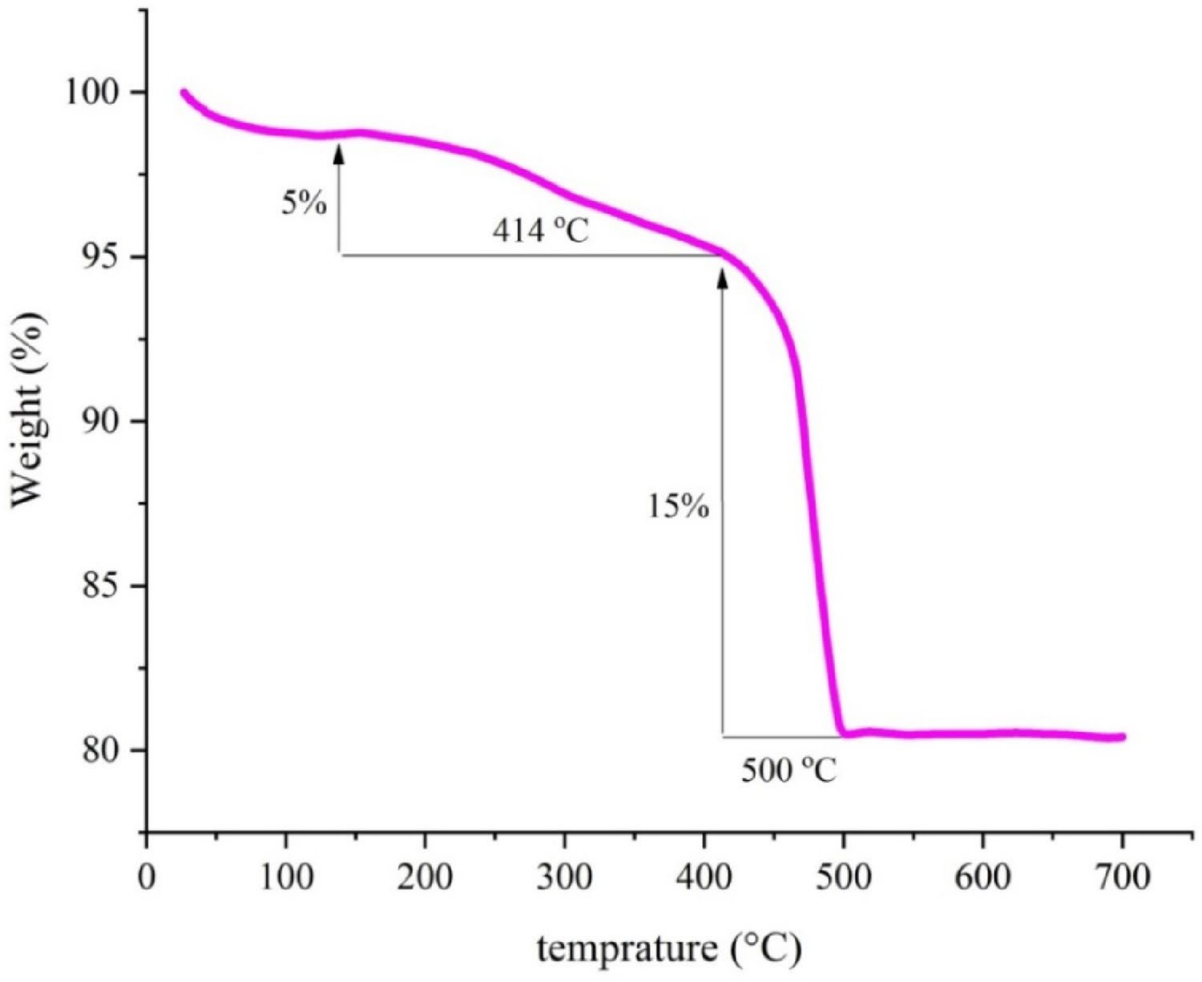
TGA of Fe_3_O_4_@SiO_2_-Im[Br]-SB-Cu (II) nano-catalyst.

The thermal stability of Fe_3_O_4_@SiO_2_-Im[Br]‐SB‐Cu (II) nano-catalyst was examined by TGA technique (Fig. 10). In the thermogram diagram of this catalyst, the maximum weight loss occurs in the range of 414–500 °C (15%), which is related to removing organic compounds from the surface of the catalyst. The 5% weight loss between 138 and 414 °C, is related to removing some organic compounds and adsorbed water molecules on the surface of iron oxide nanoparticles, respectively.

The amount of supported copper on the Fe_3_O_4_@SiO_2_-Im[Br]-SB-Cu (II) nano-catalyst was determined by AAS analysis and confirmed with ICP-OES. AAS analysis showed 0.83 mmol/g of Cu (II) on the nano-catalyst surface. This Cu(II) content was confirmed by the ICP-OES test which showed a 0.72 mmol of Cu(II) per gram of nano-catalyst.

**Figure 11:**
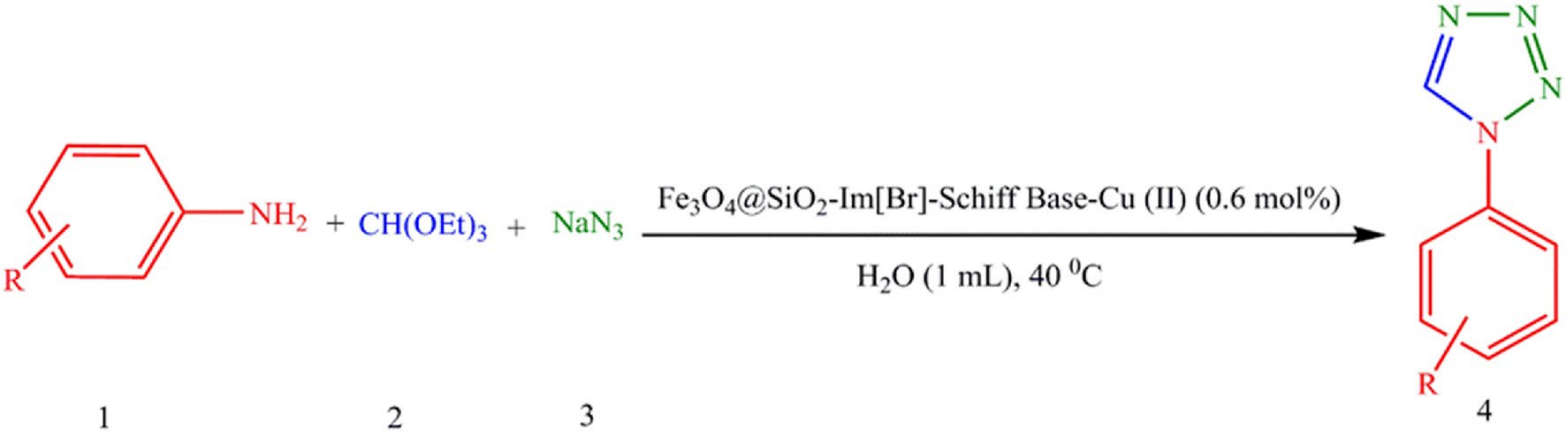
The synthesis of 1-aryl 1*H*-tetrazole derivatives.

The catalytic activity of Fe_3_O_4_@SiO_2_-Im[Br]-SB-Cu (II) was investigated in the synthesis of 1-aryl and 5-aryl 1*H*-tetrazole derivatives. The synthesis of 1-aryl 1*H*-tetrazole derivatives was optimized using the reaction model of aniline, triethyl orthoformate, and sodium azide (Fig. 11). The results of this investigation are summarized in Table 1. Firstly, the reaction efficiency in polar protic solvents (Entries 1–3) is higher than in polar aprotic and non-polar solvents (Entries 4–7). This is probably due to the ionic nature of catalyst. The reaction was run in the presence of different level of catalyst and the best result was obtained with 0.6 mol% of catalyst. A control reaction with 0 mol% of catalyst was run and as expected no product was obtained (Entry 9). Other reactions controls were tried in the presence of Fe_3_O_4_ and Fe_3_O_4_@SiO_2_-Im[Br] (Entries 14, 15) with very low efficiency. The effect of the temperature and time on the reaction were also investigated (Entries 16–21) and we concluded that the best conditions are: H_2_O solvent, 0.6 mol% of the catalyst loading, time 20 min and 40 °C. To determine the accuracy of the data generated using the small scale reported in Table 1, the best run (Entry 1) was repeated using 10 mmol scale under the same conditions and the yield of the reaction was reproducible.

**Table 1:** Optimization of the reaction conditions for the synthesis of 1-aryl 1-*H* tetrazole derivatives.

Entry	Solvent	Catalyst (mol %)	Temperature (°C)	Time (min)	Yield (%)^b^	TON^c^	TOF (h^−1^)^d^
1	**H**_**2**_**O**	**0.6**	**40**	**20**	**97**	**162**	**486**
2	MeOH	0.6	40	20	75	125	375
3	EtOH	0.6	40	20	70	117	350
4	THF	0.6	40	20	30	50	150
5	CH_3_CN	0.6	40	20	60	67	201
6	CHCl_3_	0.6	40	20	40	67	201
7	CH_2_Cl_2_	0.6	40	20	40	80	240
8	Solvent-free	0.6	40	20	85	142	426
9	H_2_O	–	40	24 h	–	–	–
10	H_2_O	0.4	40	20	80	133	399
11	H_2_O	0.9	40	20	90	150	450
12	H_2_O	1	40	20	85	142	426
13	H_2_O	1.4	40	20	78	130	390
14^e^	H_2_O	0.008 gr	40	24 h	30	50	2.08
15^f^	H_2_O	0.008 gr	40	24 h	50	83	3.5
16	H_2_O	0.6	rt	20	60	100	300
17	H_2_O	0.6	60	20	95	158	474
18	H_2_O	0.6	80	20	85	142	426
19	H_2_O	0.6	100	20	70	117	351
20	H_2_O	0.6	40	10	70	117	702
21	H_2_O	0.6	40	30	92	153	306

Significant values are in bold.

^a^Aniline (1.0 mmol), Triethyl orthoformate (1.2 mmol), Sodium azide (1 mmol) and Fe_3_O_4_@SiO_2_-Im[Br]-SB-Cu (II).

^b^Isolated yield.

^c^Turnover numbers (TONs) defined as mmol of transformed substrate molecules per mmol of catalyst.

^d^Turnover frequencies (TOFs) defined as mmol of substrate molecules transformed per mmol of catalyst per hour.

^e^Fe_3_O_4_ (0.008 gr).

^f^Fe_3_O_4_@SiO_2_-Im[Br] (0.008 gr).

After optimizing the reaction conditions, different 1-aryl 1*H*-tetrazole derivatives were synthesized by using different aniline derivatives under the same conditions (Table 2). The reaction in the presence of electron-donating and electron-withdrawing groups on benzaldehyde and the spatial barrier on aniline have significant impact on the reaction efficacy (Entries 2, 3 & 7, 8).

**Table 2:** Synthesis of 1-aryl 1*H*- tetrazole derivatives in the presence Fe_3_O_4_@SiO_2_-Im[Br]-SB-Cu (II) catalyst^a^.


Entry	R	Product	Time (min)	Yield (%)^b^	MP found (Lit.) (°C)^43^
1	H	4a	20	97	64–66 (64–65)
2	2-Cl	4b	30	86	127–130 (127–131)
3	4-Cl	4c	30	95	156–158 (157–158)
4	4-CH_3_	4d	15	92	92–94 (94–95)
5	4-OCH_3_	4e	10	92	115–117 (114–115)
6	4-NO_2_	4f	60	92	200–204 (201–202)
7	2-OH	4g	45	90	205–207 (-)
8	4-OH	4h	15	95	215–218 (-)
9	4-Br	4i	30	90	181–184 (183–185)

^a^Reaction conditions: Aniline (1.0 mmol), Triethyl orthoformate (1.2 mmol), Sodium azide (1.0 mmol) and Fe_3_O_4_@SiO_2_-Im[Br]-SB-Cu (II) (0.6 mol %) in the water at 40 °C.

^b^Isolated yield.

**Figure 12:**
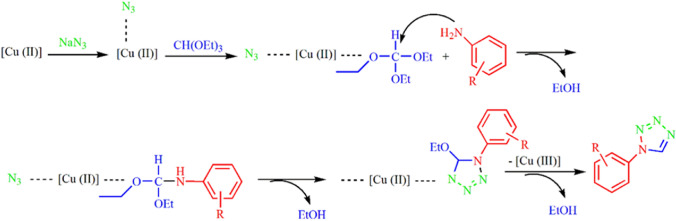
Plausible mechanism for the synthesis of 1-aryl 1*H*-tetrazole derivatives.

The plausible mechanism for the synthesis of 1-aryl 1*H*-tetrazole derivatives by using Fe_3_O_4_@SiO_2_@Im[Br]-SB-Cu (II) nano-catalyst is depicted in Fig. 12^44^. Triethyl orthoformate is activated by the N_3_-coordinated Cu(II) Nano-catalyst followed by aromatic amine attacks on the triethyl orthoformate resulting in the formation of an amide acetal intermediate. The nucleophilic attack of the azide anion on the amide acetal followed by cyclization lead to the desired tetrazole.

**Figure 13:**
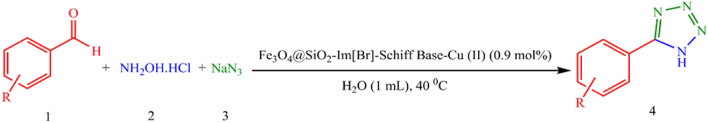
The synthesis of 5-aryl 1-*H* tetrazole derivatives.

The reaction model using benzaldehyde, hydroxy amine hydrochloride and sodium azide was selected to optimize the conditions for the synthesis of 5-aryl 1*H*-tetrazole derivatives (Fig. 13). The results of this investigation are shown in Table 3. Firstly, water was identified as the solvent of choice (Entries 1–8). As in the case of 5-aryl 1*H-*tetrazole derivatives, we confirmed that the reaction needs the catalyst to proceed and no product was obtained in the absence of the catalyst (Entries 9–13). In this reaction model, the highest conversion rate was obtained in the presence of 0.9 mol% of catalyst (Entry 1). Also in the presence of the precursor of our catalyst (Fe_3_O_4_ and Fe_3_O_4_@SiO_2_-Im[Br]), the reaction conversion rate was very low (Entries 14, 15). The effect of temperature and reaction time was also investigated (Entries 16–21) and the best conditions are: H_2_O as solvent, 0.9 mol% of the catalyst, time 20 min and 40 °C. To determine the accuracy of the data generated using the small scale reactions reported in Table 3, the best run (Entry 1) was repeated using 10 × the scale under the same conditions and the yield of the reaction (96was reproducible.

**Table 3:** Optimization of the reaction conditions for the synthesis of 5-aryl 1*H*- tetrazole derivatives^a^.

Entry	Solvent	Catalyst (mol %)	Temperature (°C)	Time (min)	Yield (%)^b^	TON^c^	TOF^d^
1	**H**_**2**_**O**	**0.9**	**40**	**20**	**97**	**108**	**323**
2	MeOH	0.9	40	20	80	88.9	267
3	EtOH	0.9	40	20	70	77.8	233
4	THF	0.9	40	20	20	22.2	66.6
5	CH_3_CN	0.9	40	20	40	44.4	133.3
6	CHCl_3_	0.9	40	20	30	33.3	100
7	CH_2_Cl_2_	0.9	40	20	30	33.3	
8	Solvent-free	0.9	40	20	70	77.8	233
9	H_2_O	–	40	24 h	–	–	–
10	H_2_O	0.4	40	20	70	140	420
11	H_2_O	0.6	40	20	85	94	282
12	H_2_O	1	40	20	90	100	300
13	H_2_O	1.4	40	20	75	83	249
14^e^	H_2_O	0.9	40	24 h	30	33.3	1.4
15^f^	H_2_O	0.9	40	24 h	50	55.5	2.3
16	H_2_O	0.9	rt	20	60	66.6	200
17	H_2_O	0.9	60	20	97	108	323
18	H_2_O	0.9	80	20	85	94.4	283
19	H_2_O	0.9	100	20	75	83.3	250
20	H_2_O	0.9	40	10	75	83	498
21	H_2_O	0.9	40	30	90	100	200

Significant values are in bold.

^a^Benzaldehyde (1.0 mmol), hydroxy amine hydrochloride (1.0 mmol), Sodium azide (1.2 mmol) and Fe_3_O_4_@SiO_2_-Im[Br]-SB-Cu (II) (0.9 mol%, 0.012 g).

^b^Isolated Yield.

^c^Turnover numbers (TONs) defined as mmol of transformed substrate molecules per mmol of catalyst.

^d^Turnover frequencies (TOFs) defined as mmol of substrate molecules transformed per mmol of catalyst per hour.

^e^Fe_3_O_4_ (0.012 gr).

^f^Fe_3_O_4_@SiO_2_-Im[Br] (0.012 gr).

Different 5-aryl 1*H*-tetrazole derivatives were synthesized using different aryl-aldehyde derivatives under the optimized conditions (Table 4). The results show that the reaction efficiency is impacted by the electronic properties and the position of the substituents groups on the benzaldehyde ring.

**Table 4:** Synthesis of 5-aryl-1*H*-Tetrazole derivatives in presence of Fe_3_O_4_@SiO_2_-Im[Br]-SB-Cu (II) catalyst^a^.


Entry	R	Product	Time (min)	Yield (%)^b^	MP found (Lit.) (°C)
1	H	4a	20	97	214–216 (215–216)^44^
2	2-Cl	4b	45	80	230–233 (-)
3	4-Cl	4c	20	80	260–262 (261–263)^45^
4	4-CH3	4d	20	82	253–254 (250–251)^48^
5	4-OCH_3_	4e	20	86	232–234 (231–232)^15^
6	4-NO2	4f	60	75	218–220 (218–219)^44^
7	2-OH	4g	30	82	221–223 (220–222)^46^
8	4-OH	4h	20	90	235–236 (233–234)^47^
9	4-Br	4i	20	92	264–266 (265)^49^

^a^Reaction conditions: Benzaldehyde (1.0 mmol), hydroxy amine hydrochloride (1.0 mmol), Sodium azide (1.2 mmol) and Fe_3_O_4_@SiO_2_-Im[Br] -SB-Cu (II) (0.9 mol %, 0.012 g) in water at 40 °C.

^b^Isolated yield.

**Figure 14:**
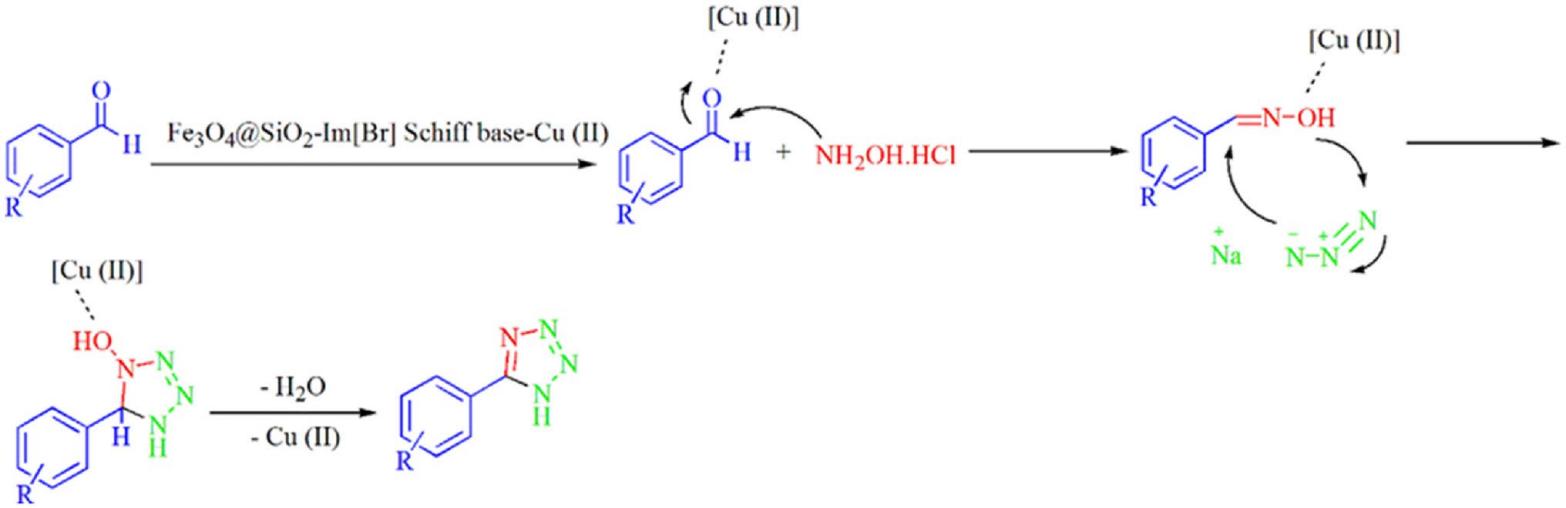
The proposed mechanism for the synthesis of 5-aryl 1*H-* tetrazole derivative.

The hypothetical mechanism for the formation of 5-aryl 1*H*-tetrazole derivatives using Fe_3_O_4_@SiO_2_-Im[Br]-SB-Cu (II) nano-catalyst is illustrated in Fig. 14^50^. The carbonyl of the aryl-aldehyde is activated by the Cu(II)-catalyst leading to the oxime formation by the attack of hydroxyl ammonium chloride. The formed Cu(II)-activated oxime reacts through a [3 + 2] cycloaddition with the azide to yield the desired 5-aryl tetrazole after elimination of a water molecule.

**Figure 15:**
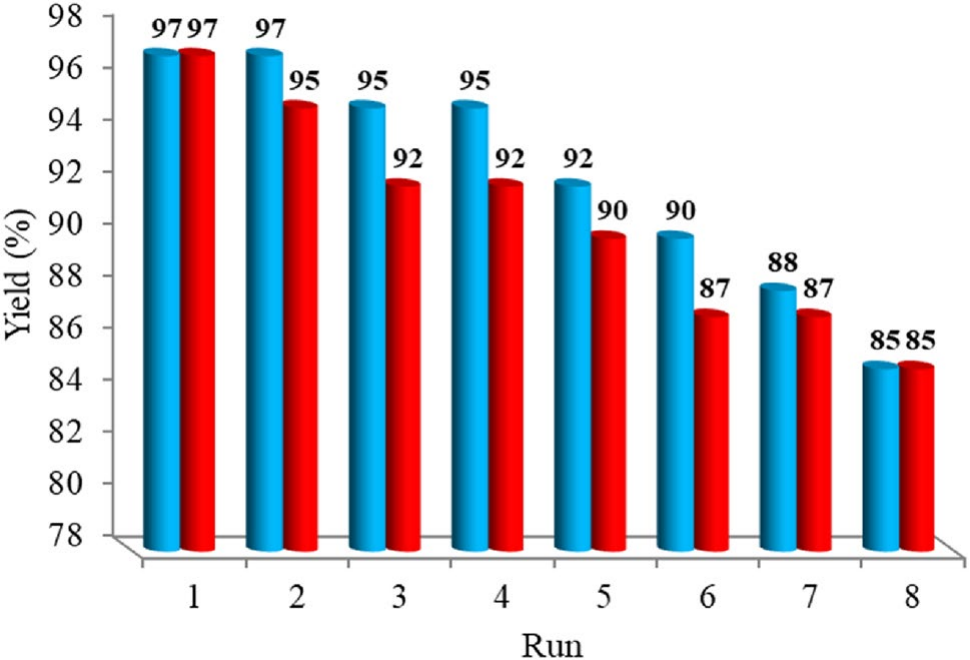
Recycling chart of Fe_3_O_4_@SiO_2_-Im[Br]-SB-Cu (II) MNP catalyst for the synthesis of 1-aryl 1*H*-tetrazole (blue) and 5-aryl 1*H*-tetrazole (red) derivatives.

**Figure 16:**
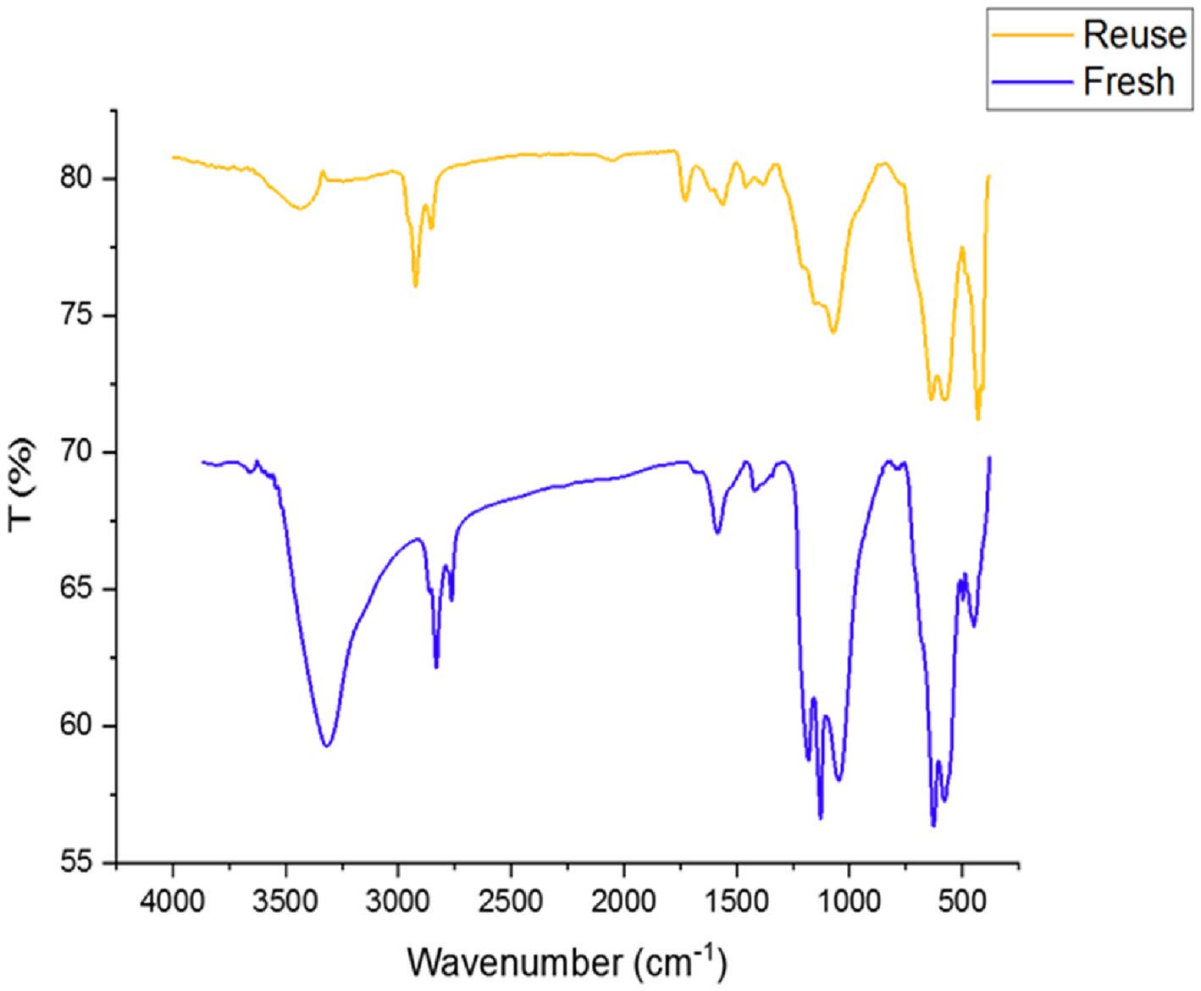
FT-IR spectra of fresh (blue) and reuse (orange) of Fe_3_O_4_@SiO_2_-Im[Br]-SB-Cu (II) nano-catalyst.

We studied the reusability of Fe_3_O_4_@SiO_2_-Im[Br]-SB-Cu (II) in the synthesis of tetrazole derivatives. After completing the reaction, the catalyst was separated by an external magnet from the reaction mixture, washed with ethyl acetate, dried, and reused in subsequent catalytic cycles under the same reaction conditions. The recycled catalyst was successfully reused for eight runs (Fig. 15) with a maximum loss of ~ 12% of yield. The FT-IR comparison of fresh and reused catalysts is shown in Fig. 16 with no change in the catalyst structure.

To investigate the heterogeneous nature of our catalyst, the hot filtration test was performed.

**Figure 17:**
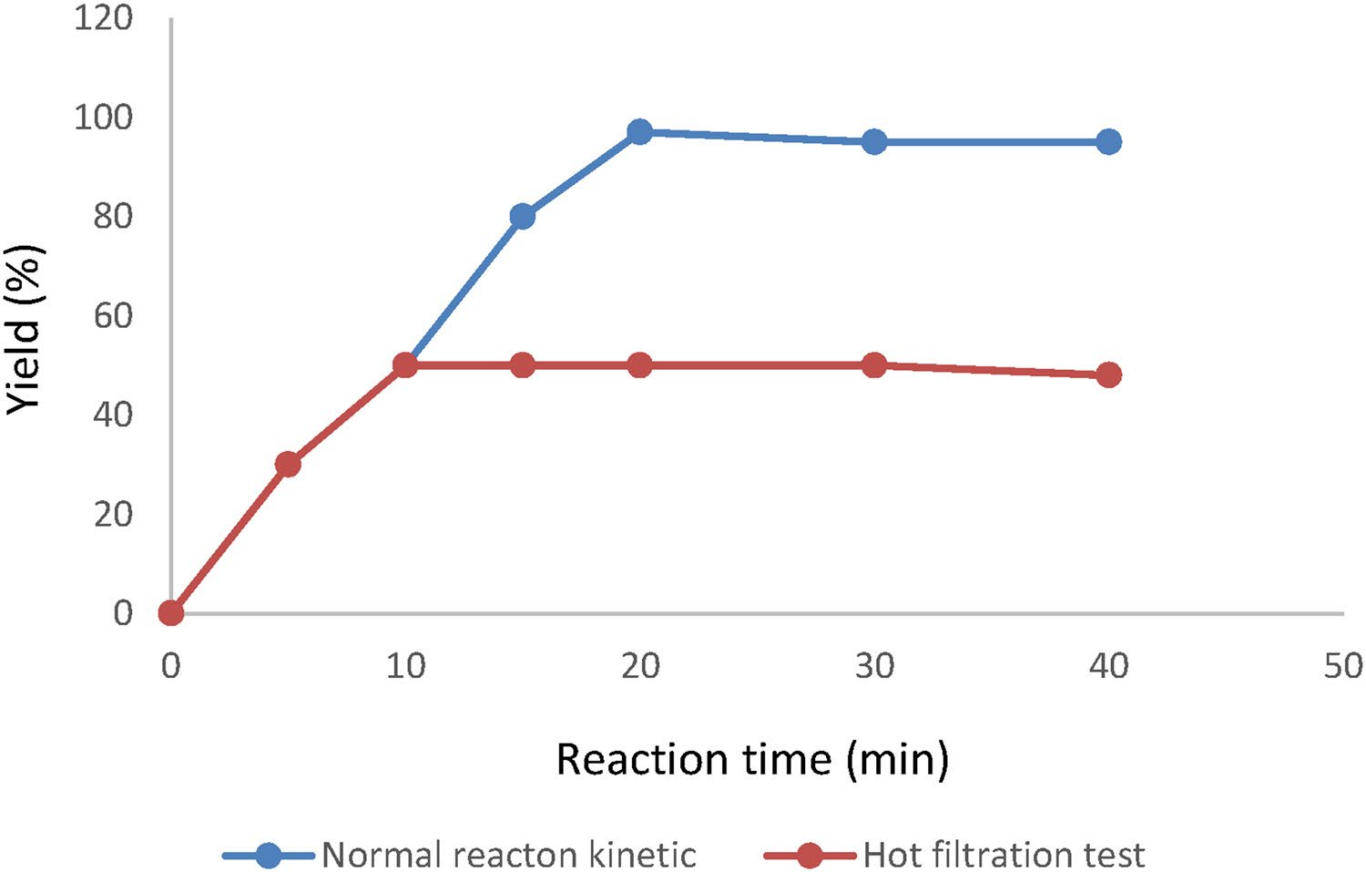
Hot filtration diagram of Fe_3_O_4_@SiO_2_-Im[Br]-SB-Cu (II) nano-catalyst in the synthesis reaction of tetrazole derivatives.

A hot filtration test was performed to evaluate the metal leaching rate and assess if the catalytic activity of our catalyst is not due to leached Cu(II) species in the reaction mixture (Fig. 17). In the model reaction for the synthesis of 1-aryl 1*H*-tetrazole derivatives after the half reaction time in which the reaction conversion rate is 50%, the reaction was stopped, and the catalyst was removed with an external magnet. The reaction mixture without the catalyst was then allowed to proceed further for 60 min. After the separation of catalyst from the reaction mixture, no increase in conversion was observed. This is a strong indication that the catalytic process is taking place only in the presence of the nano-catalyst and confirms the heterogeneity of the catalytic process. The test also indicates that there is no active copper metal species in the synthesis of tetrazole was leached into the reaction mixture.

Our catalyst was benchmarked against published catalyst for the synthesis of tetrazole derivatives (Table 5). The data in Table 5 show that our catalyst (Entry 10) is more efficient than the other reported catalysts in terms of yield and reaction time.

**Table 5:** Comparison of Fe_3_O_4_@SiO_2_-Im[Br]-SB-Cu (II) with other catalysts in synthesis of tetrazole derivatives.

Entry	Catalyst	Conditions	Time (min)	Yield (%)^a^	Refs.
1	Cu (OAC)_2_	EDS (chcl-urea), 100 °C	720	90	^51^
2	La/THH-CO_2_H@Fe_3_O_4_	PEG-600, 100 °C	300	90	^52^
3	Pd‐isatin‐boehmite	PEG-400, 120 °C	480	94	^53^
4	Fe_3_O_4_@SiO_2_-TCT-GA-Cu(II)	Ethylene glycol/H_2_O (1 : 1), 90 °C	90	97	^54^
5	PDNPs@CS-Zeo	DMF, 120 °C	720	90	^55^
6	Zn-CA-MOFs	DMF, 120 °C	480	97	^56^
7	Cu Nano-catalyst	Solvent-free, 100 °C	85	89	^57^
8	Nano-Fe_3_O_4_/In	Solvent-free, 100 °C	150	90	^58^
10	Fe_3_O_4_@SiO_2_-Im[Br]-SB-Cu (II)	H_2_O, 40 °C	20	97	This work^b^

^a^Isolated Yield.

^b^Aniline (1.0 mmol), Triethyl orthoformate (1.2 mmol), Sodium azide (1.0 mmol) and Fe_3_O_4_@SiO_2_-Im[Br]-SB-Cu (II) (0.6 mol %, 0.008 g). Benzaldehyde (1.0 mmol), hydroxy amine hydrochloride (1.0 mmol), Sodium azide (1.2 mmol) and Fe_3_O_4_@SiO_2_-Im[Br]-SB-Cu (II) (0.9 mol %, 0.012 g).

## Conclusion

We have reported the preparation of novel heterogeneous recoverable and reusable nano-catalyst, Fe_3_O_4_@SiO_2_-Schiff base-Cu(II) complex, which is able to catalyze green formation of 1- and 5-substituted 1*H*-tetrazoles using multicomponent reaction (MCR) approach between aromatic amines, ethyl orthoformate and sodium azide for the preparation of 1-aryl 1*H*-tetrazole derivatives and between aryl-aldehydes, hydroxylamine hydrochloride and sodium azide for the synthesis of 5-aryl 1*H*-tetrazole under mild conditions and short reaction time in water. The catalyst was well characterized by various techniques including FT-IR, VSM, XRD, EDX, FE-SEM, TEM, TGA, ICP and AAS. This nano-catalyst simplicity, high efficiency, convenient reusability, ease of work-up are among its critical advantages. Various aromatic aldehydes and aromatic amines served as suitable substrates for the preparation of substituted tetrazoles via MCR protocol with high to excellent yields. This nano-catalytic system has undeniably proven as an efficient and ecofriendly catalyst for aryl-substituted tetrazoles synthesis. Future investigation in the synthesis of alkyl-substituted tetrazoles using aliphatic aldehydes and amines will determine the scope of this nano-catalyst application.

## Data Availability

The datasets generated and/or analyzed during the current study are not publicly available due to the continuation of the work at Faculty of Science, University of Birjand, Iran, but are available from the corresponding author on reasonable request.
